# The Romance of Camphor

**Published:** 1914-06-13

**Authors:** 


### The Romance of Camphor.

In a most interesting paper the Editor of the *Journal of Tropical Medicine and Hygiene* describes the astounding conditions in which camphor is obtained in the island of Formosa, the principal source of the world's supply of this pungent drug. Although naphthalene and other products may have somewhat ousted camphor from the institutional linen room and bedding store, the institutional dispensary still relies on the drug for the compounding of many liniments and other galenical preparations. It appears that the camphor tree grows only in the most mountainous parts of Formosa, inhabited by head-hunting savages, whom none of the successive invaders of the island have been able to subdue. These savages are quite alive to the value of the trees, and fiercely oppose all attempts to get possession of the forests. Since the Japanese took the island after their successful campaign against China in the 'nineties they have been carrying on a carefully conceived plan of gradual penetration. They make paths six feet in width through the virgin forests; every 120 yards stands a guard-house, and every fourth or fifth guard-house is a small fort, entrenched and defended by wire entanglements. Telephonic intercommunication, machine guns, and all the resources of Western military science are employed; and the lines are pushed gradually forward. In spite of these elaborate precautions, the loss among the camphor gatherers amounts to hundreds of deaths annually. It is calculated that Formosa contains about one million camphor trees, some ten thousand of which are cut down every year. At this rate the supply will be exhausted in a hundred years; but when the country is thoroughly pacified there is no doubt that the Japanese will see that reafforestation is properly undertaken and an inexhaustible supply ensured. The savages are estimated to number about 120,000; and a further twelve years will, it is thought, be required to subdue them. He concludes that the popular saying as to every particle of camphor having cost its weight in blood has some measure of justification. Next time the institutional physician orders compound tincture of camphor in a cough mixture, let him reflect on this!

